# Drugs-Induced Pathological Gambling: An Analysis of Italian Spontaneous Reporting System

**DOI:** 10.1007/s10899-019-09828-1

**Published:** 2019-01-23

**Authors:** Cristina Scavone, Barbara Stelitano, Concetta Rafaniello, Francesco Rossi, Liberata Sportiello, Annalisa Capuano

**Affiliations:** Department of Experimental Medicine Section of Pharmacology “L. Donatelli”, University of Campania “Luigi Vanvitelli”, Campania Regional Centre for Pharmacovigilance and Pharmacoepidemiology, Via Costantinopoli 16, 80138 Naples, Italy

**Keywords:** Pathological gambling, Pharmacovigilance, Italian spontaneous reporting system, RNF

## Abstract

Pathological gambling has been reported as a direct complication of Parkinson’s disease and its pharmacological treatment based on dopamine agonists. Moreover, further medications (not dopamine agonists) were associated to the occurrence of gambling disorder. We aim to analyze the spontaneous reports of gambling disorder on the whole Italian territory with a focus on Campania Region (Southern Italy) from January 1st 2002 to July 31st 2018. We analyzed gambling disorder’s reports across the 2002–2018 period in the Italian spontaneous reporting database (Rete Nazionale di Farmacovigilanza—RNF), with a focus on Campania region. 94 suspected cases of gambling disorder associated to apomorphine, aripiprazole, cabergoline, levodopa, levodopa and derivatives in association with entacapone/benserazide and carbidopa, pergolide, pramipexole, ropinirole, and rotigotine were reported into the RNF. Of these cases, two related to pramipexole and one to aripiprazole were sent to Campania Pharmacovigilance Regional Centre. Although it is widely recognized that dopamine agonists may induce behavioral disorders, Parkinson’s disease is itself associated to pathological gambling, compulsive shopping and eating. Since our results could not clarify the correlation between Parkinson’s disease, its pharmacological treatment and pathological gambling, in order to better define this correlation there is a need to conduct further ad hoc observational studies.

## Introduction

During the last few years, the availability of legal gambling has abruptly increased leading to a huge intensification in pathological gambling (Potenza et al. 2002). Among European countries, Italy holds the absolute record for gambling related receipt. In 2016 the gaming sector has brought almost 10 billion euros, equal to 0.6% of the Italian gross domestic product (Fisco, entrate record dal gioco d’azzardo: in Europa nessuno come noi. Available at: http://www.today.it/economia/gettito-fiscale-giochi-scommesse-italia-dati.html).

According to the fifth edition of the Diagnostic and Statistical Manual (DSM-5), gambling disorder is defined as a persistent and recurrent behavior leading to clinically significant impairment or distress. Patients affected by gambling disorder present at least four of the following attitudes or feelings: they need to gamble with increasing amounts of money; they present restlessness or irritability when stop or reduce gambling; they have made several failed efforts to control or stop gambling; they are worried about gambling; they gamble during anxious or depressed situations; they gamble despite loss of money; they lie about the extent of involvement with gambling; they usually lost any relationship, job, or career opportunity due to gambling; they rely on others to provide money (DSM-5 Diagnostic Criteria: Gambling Disorder. Available at: http://www.ncpgambling.org/wp-content/uploads/2014/08/DSM-5-Diagnostic-Criteria-Gambling-Disorder.pdf). Several risk factors have been identified in inducing gambling disorders, including biological ones, such as changes in dopamine and serotonin neurotransmission, genetic, psychological and social factors (Fong 2005; Comings et al. 1996).

In recent years, pathological gambling has also been reported as a direct complication of Parkinson’s disease and its pharmacological treatment based on dopamine agonists (Seedat et al. 2000; Weintraub and Claassen 2017). Following an evaluation performed by the EU Pharmacovigilance Working Party, the Italian Medicine Agency (AIFA) released a public statement in which announced an increased risk of impulse control disorders, including pathological gambling, increased libido, compulsive buying and eating, in patients treated with dopamine agonists for Parkinson’s disease, restless legs syndrome and endocrine disorders (Agenzia Italiana del Farmaco. Importanti informazioni sulla sicurezza riguardanti i farmaci dopamino agonisti. Available at: http://www.agenziafarmaco.gov.it/wscs_render_attachment_by_id/111.71353.11724881557268b10.pdf?id=111.71359.1172488156158; PhVWP monthly report on safety concerns, guidelines and general matters. http://www.ema.europa.eu/docs/en_GB/document_library/Report/2012/07/WC500130391.pdf). On the basis of new evidence, sections “Special warnings and precautions for use” and “Adverse effects” of the Summary of product Characteristics (SPCs) of apomorphine, bromocriptine, cabergoline, alpha-dihydroergocryptine, lisuride, pergolide, piribedil, pramipexole, quinagolide, ropinirole, levodopa and derivatives in association with entacapone/benserazide and carbidopa, were modified highlighting the new risk of impulse control disorders (Peterson and Forlano 2017; Mété et al. 2016). Moreover, further medications, including aripiprazole, modafinil, rotigotine, sertraline, citalopram, and lamotrigine, were associated to the occurrence of gambling disorder (George et al. 2015; Schreglmann et al. 2012; Ramasubbu 2004).

Given the clinical relevance of pathological gambling, the safety warning issued by European regulatory agencies about the increased risk of this adverse drug reaction (ADR) in patients treated with dopamine agonists and the literature data that suggested the same risk also with further medications, we aim to analyze the spontaneous reports of gambling disorder on the whole Italian territory with a focus on Campania Region from January 1st 2002 to July 31st 2018.

## Methods

### Data Source

The Italian Pharmacovigilance System is coordinated by the AIFA, that established in 2001 a National Pharmacovigilance Network (Rete Nazionale di Farmacovigilanza, RNF) for the collection of individual case safety reports (ICSRs). The AIFA works in collaboration with Pharmacovigilance Regional Centers (Mazzitello et al. 2013), which are involved in the evaluation of ICSRs, in terms of quality of data, evaluation of causality assessment for each drug-vaccine/ADR couple, and participation to signal analysis on drugs and vaccines.

In accordance with Italian pharmacovigilance regulations, ICSRs entry into the RNF at national level can be shared only as pooled data through the RAM system, while ICSRs entry into the RNF at regional level are accessible to the Pharmacovigilance Regional Center of competence directly through the RNF. Information are more refined in the latter case.

For the purpose of this study, we retrieved from the RAM system (for Italian safety data) and the RNF (for regional safety data) all ICSRs that reported gambling disorder as ADR and apomorphine, bromocriptine, cabergoline, alpha-dihydroergocryptine, lisuride, pergolide, piribedil, pramipexole, quinagolide, ropinirole, levodopa and derivatives in association with entacapone/benserazide and carbidopa, aripiprazole, modafinil, rotigotine, sertraline, citalopram or lamotrigine as the suspected drug.

### Descriptive Analysis and Case-Series

All ICSRs, reported from January 1st 2002 to July 31st 2018, that described the occurrence of gambling disorder related to the previous mentioned suspected drugs were selected.

For ICSRs reported on the whole Italian territory we performed a descriptive analysis in terms of number of gambling disorder’s reports. For ICSRs reported in Campania region, we performed a descriptive analysis of gambling disorder’s reports, stratifying by suspected drug (s), concomitant drug (s), age, gender, seriousness and outcome degrees, and causality assessment. Regarding to the seriousness degree, ADRs are classified as serious, when they induce death, hospitalization or prolongation of hospitalization, severe or permanent disability, life-threat, congenital abnormalities/birth deficits, or if they were considered clinically relevant, or not serious. The outcome is categorized as: completely resolved, resolved with sequelae, improved, unchanged and unavailable (Sessa et al. 2016a, b). The causality assessment was performed through Naranjo algorithm (Naranjo et al. 1981; Mascolo et al. 2017) in order to establish the strength of relationship between the drug and the ADR. All scores ranged between possible and certain reports were considered reasonable for causality.

### Compliance with Ethical Standards

Safety data deriving from the Italian spontaneous reporting system are anonymous and in compliance with ethical standard. Therefore, no further ethical measures were required.

## Results

### Gambling Disorder in Italy

**Fig. 1:**
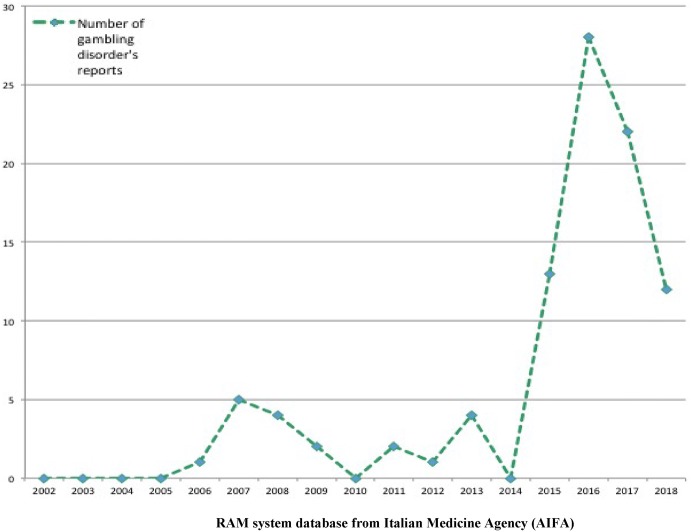
Trend in gambling disorder’s reporting in Italy (period: 2002–July 2018)

**Fig. 2:**
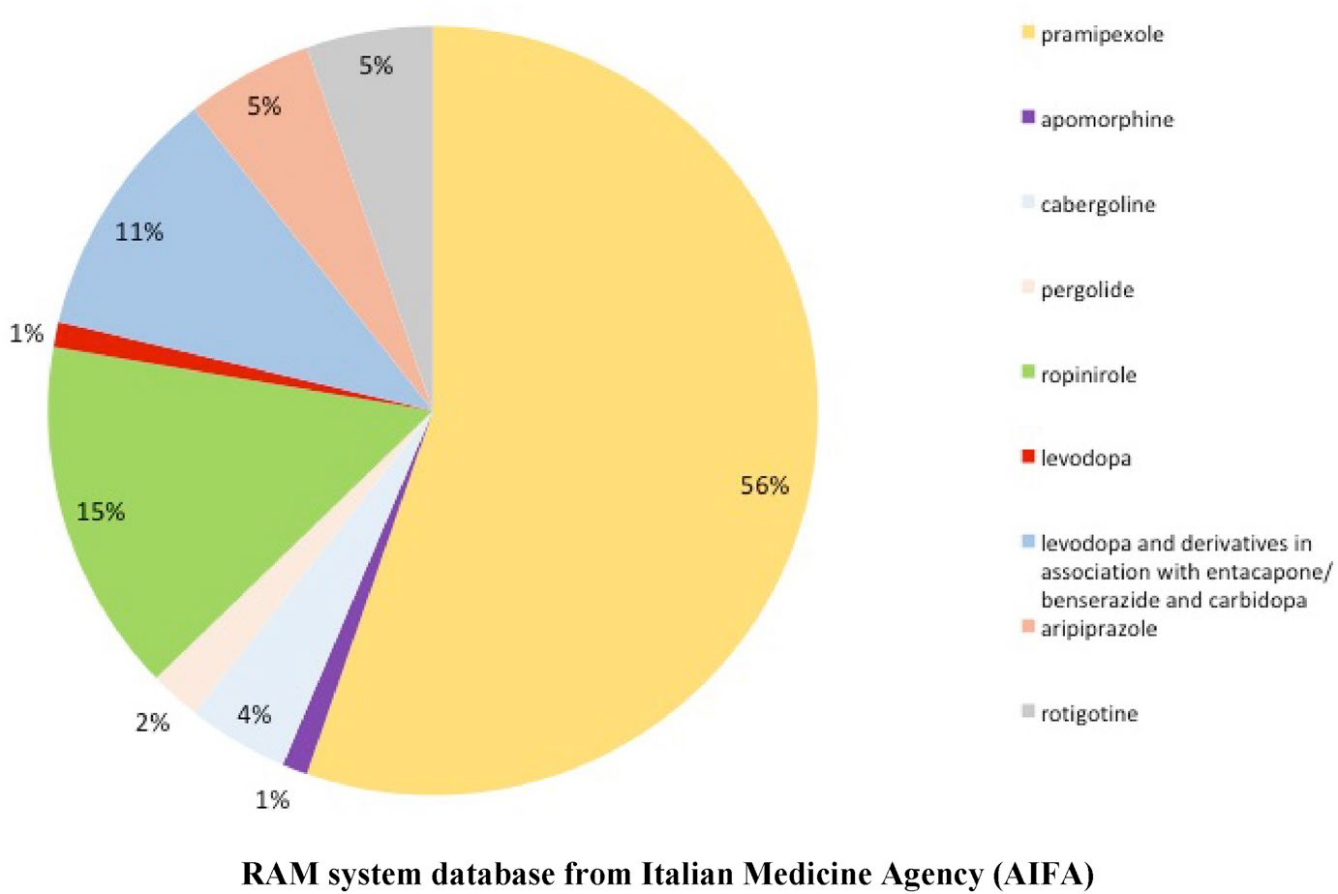
Distribution (%) by suspected drug in gambling disorder’s reports in Italy (period: 2002–July 2018)

From January 1st 2002 to July 31st 2018, 94 suspected cases of gambling disorder associated to apomorphine, aripiprazole, cabergoline, levodopa, levodopa and derivatives in association with entacapone/benserazide and carbidopa, pergolide, pramipexole, ropinirole, and rotigotine were reported into the RNF and listed in the RAM system. As shown in Fig. 1, from 2006 until 2016 the reporting of drug-induced gambling disorder had gradually increased, with a fluctuating trend. More than 80% of gambling disorder’s reports were related to pramipexole (n = 52; 56%), ropinirole (n = 14; 15%), and levodopa in association with entacapone/benserazide and carbidopa (n = 10; 11%) (Fig. 2). No cases of gambling disorder were reported for bromocriptine, alfa-dihydroergocryptine, lisuride, piribedil, quinagolide, modafinil, sertraline, citalopram and lamotrigine.

### Gambling Disorder in Campania Region

Out of 94 suspected cases of gambling disorder reported in Italy from January 1st 2002 to July 31st 2018, 3 were sent to the Campania Pharmacovigilance Regional Centre and reported into the RNF (Table 1). No ICSRs that reported gambling disorder as ADR and apomorphine, bromocriptine, cabergoline, alpha-dihydroergocryptine, lisuride, pergolide, piribedil, quinagolide, ropinirole, levodopa and derivatives in association with entacapone/benserazide and carbidopa, modafinil, rotigotine, sertraline, citalopram or lamotrigine as the suspected drug were sent to the Campania Pharmacovigilance Regional Centre. Among the three cases sent to the Campania Pharmacovigilance Regional Centre, two were related to pramipexole, one of which associated to domperidone, and one to aripiprazole. No drugs other than dopamine modulators were involved as suspected. In none of these cases, gambling disorder was classified as serious. A description of those cases is set out below.

**Table 1:** Main features of gambling disorder’s reports in Campania Region (period: 2002–July 2018)

Age (years)	Gender	Suspected drugs	Concomitant drugs	Seriousness	Outcome
56	M	Pramipexole	Amantadine	Not serious	Completely resolved
			Levodopa/carbidopa		
			Clonazepam		
54	M	Pramipexole domperidone	–	Not serious	Not available
32	F	Aripiprazole	Delorazepam	Not defined	Not available
			Alprazolam		

RNF database from Campania Region, Southern Italy

#### Case One

A 56-year-old male patient was receiving pramipexole (0.7 mg/day) for the treatment of Parkinson’s disease when he presented gambling disorder. The patient was concomitantly receiving amantadine (200 mg/day), levodopa/carbidopa (500 mg/day), and clonazepam. Positive dechallenge was reported for this patient, while no information is available on the rechallenge. The latency period elapsed between the first exposure to pramipexole and the diagnosis of gambling disorder was 1 week. The evaluation of causality assessment between pramipexole and the study event resulted in possible.

#### Case Two

A 54-year-old male patient was receiving pramipexole (unknown dose) and domperidone (unknown dose) when he presented gambling disorder. No information is available on dechallenge, rechallenge, and on the latency period elapsed between the first exposure to the suspected medicine and the diagnosis of gambling disorder. The evaluation of causality assessment between pramipexole and domperidone and the study event resulted in possible.

#### Case Three

A 32-year-old Caucasian female patient was receiving aripiprazole (5–10/day/orally) for the treatment of mood disorder when she experienced gambling disorder. The patient was also receiving an intramuscular long-acting injectable depot formulation of aripiprazole (also indicated as suspected drug) for an unknown therapeutic indication, and two concomitant drugs, delorazepam and alprazolam. Positive dechallenge was not reported for this patient and no information is available on the rechallenge. The latency period elapsed between the first exposure to suspected medicines and the diagnosis of gambling disorder was 4 months. The evaluation of causality assessment between aripiprazole and the study event resulted in possible.

## Discussion

Our results demonstrated that, from 2002 until July 31st 2018, 94 ICSRs describing the occurrence of gambling disorders were reported into the RNF on the entire Italian territory. Drugs most commonly related to gambling disorder were pramipexole, listed as suspected drug in 56% of all ICSRs, ropinirole in 15% of all ICSRs, levodopa in association with benserazide/entacapone and carbidopa in 11%, aripiprazole and rotigotine, each one in 5%. Out of 94 ICSRs reported on the whole Italian territory, 3 were reported to the RNF in Campania Region. Such reports referred to the occurrence of gambling disorder in patients treated with pramipexole (one of which associated to domperidone) and aripiprazole. Although there is no reasonable explanation for a so limited number of gambling disorder cases related to drugs evaluated in our study in Campania Region, it should be highlighted that, according to the data recently reported by the AIFA, the most commonly used drugs in our Region are those with ATC code A, C, J or R and not ATC N drugs (subjects of our study) (L’uso dei farmaci in Italia. Rapporto Nazionale anno 2017. http://www.aifa.gov.it/sites/default/files/Campania-Uso_dei_farmaci_nel_2017.pdf).

At present, several studies have confirmed a strict correlation between dopamine agonists and pathological gambling. A cross-sectional study, which enrolled 3090 patients diagnosed with Parkinson’s disease and treated with either levodopa or a dopamine agonist, found that those drugs were associated with a 2- to 3.5-fold increased risk of presenting an impulse control disorder (Weintraub et al. 2010). Furthermore, according to Santangelo et al. (2013) the prevalence of pathological gambling is 2.2–7% in patients with Parkinson’s disease receiving medications. In line with our results, several case reports described the occurrence of gambling disorder in patients treated with pramipexole for the treatment of Parkinson’s disease, restless legs syndrome or bipolar disorder. In these cases, the time of ADR occurrence from the first administration of pramipexole or other dopamine agonists ranged from 1 to 10 months (d’Orsi et al. 2011; Kolla et al. 2010; Strejilevich et al. 2011). The possible explanation of pramipexole-induced pathological gambling has to be found in its pharmacodynamic properties. Indeed, pramipexole is a dopamine agonist, relatively selective for D3 receptors, which are mainly located in the mesolimbic pathways where cognitive and emotional functions, including pleasure and addiction, are overseen. Apart from limbic areas, D3 receptors are also co-expressed with D2 in sensory thalamic nuclei, mammillothalamic tract, amygdala, and therefore they play a key role in controlling physiologic and sensitive aspects of novelty and reward (Kelley et al. 2012). The same pharmacodynamic properties are also shared by ropinirole and rotigotine (Seeman 2015), both associated to the occurrence of impulse control disorders in our study. Post-marketing safety data showed that the most commonly reported ADRs with ropinirole are hypersensitivity, somnolence and psychotic reactions, including pathological gambling (Stocchi et al. 2014). Similarly, rotigotine was associated to the occurrence of impulse control disorders in three patients with Parkinson’s disease (Wood et al. 2015; Wingo et al. 2009). All of them were concomitantly receiving further medications, including levodopa, entacapone, amantadine, and selegiline. In all patients, gambling disorder improved with the reduction in rotigotine dose or its discontinuation. Likewise, cabergoline and pergolide are ergot derivatives that act as dopamine agonists with highest affinity for D2 and D3 receptors; cabergoline is also a 5-HT receptors agonist (Leggio et al. 2016). Two case reports describing the correlation between these ergot derivatives and pathological gambling are reported in the literature. The first one refers to a 46-year-old man who developed gambling disorder after the initiation of cabergoline for the treatment of prolactinoma (Gahr et al. 2011), while the second one refers to a 42-year-old man who developed gambling disorder during treatment with pergolide and levodopa for Parkinson’s disease (Larner 2006).

Though it seems that D3 agonists may be preferentially related to gambling and similar behavioral disorders, recent literature data have suggested a correlation also between this ADR and aripiprazole, which is an atypical antipsychotic, that acts as a partial agonist at D2 receptors and 5-HT1a and 5-HT2 serotonin receptors (Khanna et al. 2014). Therefore, aripiprazole could induce gambling due to its agonist activity in the mesocortical pathway, where a low dopamine activity seems to be related to cognitive changes (Gavaudan et al. 2010). In this respect, several case reports have described the occurrence of gambling disorder in patients diagnosed with mood, depressive and schizoaffective disorder and treated with aripiprazole. Most of these cases occurred from few days to one year since the initiation of aripiprazole treatment and tended to resolved after drug’s discontinuation (Gaboriau et al. 2014; Smith et al. 2011; Cohen et al. 2011). According to the results of a retrospective analysis of reports sent to the FDA Adverse Event Reporting System from 2003 to 2012, 1580 impulse control disorders cases were identified. Among suspected drugs, dopamine agonists, including pramipexole and aripiprazole, showed the strongest association with impulse control disorders (Moore et al. 2014). Similar results were obtained from a cohort study conducted on the health claims LifeLink database (Etminan et al. 2017).

In our study, few cases of gambling disorder were related to levodopa, levodopa in association with decarboxylase inhibitors or entacapone, and apomorphine. Levodopa has been used for the treatment of Parkinson’s disease for over 50 years. It is a dopamine precursor that passes the blood–brain barrier, normally administered in combination with decarboxylase inhibitors (benserazide/carbidopa), which increase levodopa brain concentration, tolerance and clinical efficacy (LeWitt 2015) and with entacapone, a monoamine oxidase inhibitor, that improve wearing off symptoms (Kouppamäki et al. 2015). According to literature data, both levodopa and apomorphine were associated to gambling disorder (Symmonds et al. 2013; Pontieri et al. 2015; Boyle and Ondo 2015; van Eimeren et al. 2010).

Among ICSRs reported into the RNF in Campania region, patients were concomitantly administered other drugs, including benzodiazepines. Although such medicines do not seem directly related to the occurrence of gambling disorder, benzodiazepines, together with amphetamines, methylphenidate, and hydrocodone, are frequently used as cognitive and performance enhancing medications among poker players (Caballero et al. 2016), suggesting in our opinion a possible role in increasing the degree of gambling. Moreover, among our cases, patients who experienced gambling disorder were concomitantly receiving other drugs, including amantadine and domperidone. Although amantadine is associated with impulse control disorders (Thomas et al. 2010), currently data are conflicting. In fact, according to Pettorruso et al. amantadine seems to be an efficacious treatment of pathological gambling, leading to a reduction in severity of gambling symptoms. Authors have suggested that these effects are related to the ability of amantadine to interact with glutamate homeostasis and dopamine function which lead to a reduction in gambling craving and behavior (Pettorusso et al. 2012). Finally, to our knowledge, no data supporting the association domperidone/gambling disorder exists; nevertheless, since this drug is a peripheral D2 receptor antagonist with a low pass through the blood–brain barrier, we should suppose that domperidone-induced gambling disorder is very rare (Barone 1999).

In conclusion, although it is widely recognized that dopamine agonists may induce behavioral disorders, it should be noted that Parkinson’s disease is itself characterized by several non-motor symptoms which include pathological gambling, compulsive shopping and eating. In this context, the dysregulation of the dopamine system/dopamine receptor functionality could possibly represent the main neurobiological mechanism underlying the association pathological gambling/Parkinson’s disease, although alterations in serotonin and opioid transmission cannot be excluded (Weintraub and Claassen 2017; Majuri et al. 2017). In particular, literature data have suggested that gambling occur in approximatively 1.7–6.1% of patients with Parkinson’s disease (Balconi et al. 2018). Those symptoms usually occur in the early stages of disease and negatively affect patients’ quality of life (Manning et al. 2015). Similarly, patients with mood and bipolar disorder are more likely to suffer of any form of addiction, including pathological gambling (Voon et al. 2006; Jones et al. 2015).

## Strengths and Limitation

This is an analysis of a spontaneous reporting data, which are limited by several factors, such as under-reporting, flexible quality of data and lack of information. As a matter of fact, ICSRs related to cases of gambling disorders sent to the RNF in Campania region lacked essential clinical information, such as those related to dechallenge, rechallenge, date of ADR’s occurrence and/or starting date of therapy, which inevitably affected the proper evaluation of causality assessment. Furthermore, we cannot rule out the presence of other possible confounding variables that might have contributed to the occurrence of gambling disorders, such as a history of gambling or comorbidities not reported in ICSRs. Furthermore, other factors which could trigger the development of the study event are concomitant medications. Indeed, two out of three patients who experienced gambling disorder in Campania region were concomitantly receiving amantadine and benzodiazepines. Therefore, we cannot rule out the influence of concomitant drugs on the development of clinical symptoms. Lastly, it should be take into account difficulties in identifying gambling disorder as an ADR instead of a social attitude.

Despite limitations that affect the spontaneous reporting system, it is largely accepted that this pharmacovigilance method is a simple and inexpensive tool, that allows to detect rare and serious ADRs not identified during premarketing clinical trials (Auricchio et al. 2017), such as gambling disorder. Furthermore, this method allows to generate safety hypothesis on medicines, that shall be confirmed or refuted by ad hoc pharmacovigilance studies.

## Conclusion

In an attempt to describe the occurrence of drug-induced gambling disorders in a real life setting, we analyzed data reported into the RNF in Italy, with a focus on Campania Region, from 2002 to July 31st 2018. We found that during 17 years of observation 94 suspected cases of gambling disorder were reported on the whole Italian territory and among these three cases of dopamine agonists-induced gambling disorder were reported in Campania. In our view, our results represent the consequences of an effective risk communication procedures made by the European Medicine Agency in this field.

As recommended in dopamine agonists’ SPCs, a higher control of patients for the development of impulse control disorders and eventually the dose reduction or discontinuation of dopamine agonists could represent an effective strategy to reduce the occurrence of pathological gambling and its negative impact. Therefore, the close monitoring of patients treated with dopamine agonists or previous mentioned drugs for the early identification of such ADR could improve quality of life of patients and their caregiver by reducing the social negative impact of pathological gambling both for the patient and his family. Since literature data and those obtained from the Italian spontaneous reporting system did not clarify the correlation between Parkinson’s disease, its pharmacological treatment and pathological gambling neither between the other drugs that we have investigated and pathological gambling, further ad hoc observational studies, which are important to allow the early detection of unexpected and/or serious ADRs (Capuano et al. 2014; Scavone et al. 2017), should be specifically planned.
